# Photo-curable poly-(ethylene glycol)–fumarate elastomers with controlled structural composition and their evaluation as eluting systems

**DOI:** 10.1039/c8ra09336a

**Published:** 2019-01-02

**Authors:** Lucila Navarro, Roque J. Minari, Santiago E. Vaillard

**Affiliations:** Instituto de Desarrollo Tecnológico para la Industria Química (INTEC), CCT-Santa Fe CONICET-UNL. Colectora Ruta Nacional 168, Km 1, Predio “Dr. Alberto Cassano”, Paraje “EL Pozo” Santa Fe 3000 Argentina svaillard@intec.unl.edu.ar

## Abstract

Elastomeric poly-ester materials have extraordinary potential for soft tissue engineering applications. In connection, in the last 10 years, cross-linkable oligo-(polyethylene glycol fumarate)s emerged as promising materials for obtaining hydrogels for bone tissue engineering applications. In this work we prepared a new family of photo-curable poly-(ethylene glycol)–fumarate elastomers with controlled structural composition. These novel elastomers were obtained by photo-curing of fumarate pre-polymers based on diethylene glycol and oligo-ethylene glycols (PEGs 200 and 400), under extremely mild experimental conditions using a low power UV source. The synthesis of fumarate pre-polymers, which were obtained by thermal poly-condensation, and the photo-curing process, were both here discussed on the basis of their structural differences and proposed operating mechanisms. Finally, the photo-radical cross-linking reactions were performed in the presence of anti-cancer drugs (doxorubicin and paclitaxel), in order to evaluate the potential application of the elastomers as new eluting systems. Thus, different release profiles were obtained for hydrophilic (doxorubicin) and hydrophobic (paclitaxel) anticancer drugs, and these differences are discussed on the basis of the structure of the elastomers.

## Abstract 2

Elastomeric poly-ester materials have extraordinary potential for soft tissue engineering applications.

## Graphical abstract

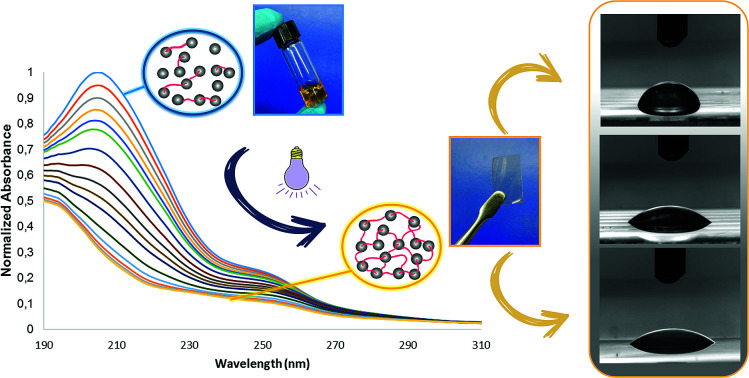

## Introduction

The preparation of new synthetic polyester materials, with tailored mechanical properties and degradation profiles, has been crucial for the development of tissue engineering approaches for regenerative medicine and for the constructions of new drug release systems for cancer treatment.

The radical mediated cross-linking reactions of (met)acrylic acid esters and related compounds are well known processes, traditionally used for the preparation of thermosets and various resins.^1^ Related α,β-unsaturated carboxylic acids, such as fumaric and maleic acid, have also been used for the preparation of resins and thermosets. Given that the structure of these monomers can lead to unfavorable curing kinetics, they are usually co-polymerized with other vinyl monomers.^2^

Poly-(ethylene glycol) (PEG) is an important hydrophilic polymer of intense application for the improvement of the therapeutic properties of labile peptide and protein bio-pharmaceuticals (PEGylation technology). Among the various PEG-based materials that have been published, most of the attention has been focused PEG–fumarate hydrogels,^3–5^ and other cross-linked PEG–fumarates, such as PEG–fumarate cross-linked with PEG-diacrylate;^6^ all of which appear as extremely promising candidates for bone tissue engineering scaffolds. Some examples of the potential of PEG–fumarate hydrogels include the encapsulation of marrow stromal and pigment epithelial cells,^7,8^ the modification of the gel with arginyl-glycyl-aspartic acid (RGD peptide)^9^ and several other uses, including the controlled release of gentamicin, proteins and growth factors.^10–14^

PEG–fumarate is usually obtained by the reaction of fumaryl chloride with PEG using triethylamine as HCl scavenger,^15^ followed by thermal or light induced cross-linking to yield the required hydrogel. An alternate method, which involves the DDC-DMAP mediated esterification of fumaric acid and PEG, has also been developed.^16^

The use of elastomers for the controlled release of drugs has barely been reported, probably because the preparation of the drug eluting elastomeric systems usually require a thermal curing step at high temperature. Therefore, only drugs with extremely high thermal resistance and without chemical groups that could react with the polymer can be loaded to the polymeric matrix before the release. On the other hand, photo-crosslinkable elastomers would offer the possibility of loading a drug to the pre-polymer and cross-linking could be ideally performed directly by *in situ* UV light exposure over the tumor. In this matter, despite of the standalone relevance of PEG–fumarate hydrogels, the potential of these pre-polymers for the preparation of elastomeric eluting systems for drug delivery has so far not been reported. The main goals of this work are to investigate the synthesis of PEG–fumarate elastomeric materials with controlled structure composition and to explore their potential as novel anticancer drugs delivery systems. Therefore, we synthesized PEG–fumarate pre-polymers by melt thermal poly-condensation of fumaric acid and ethylene glycol, di-ethylene glycol and PEGs of 200 and 400 Da. Then, the pre-polymers were cross-linked to obtain elastomeric networks by irradiation with a low power UV light source, under mild conditions, in the presence of a suitable radical initiator (Irgacure 500). Finally, we evaluated the potential of the materials for the construction of new eluting systems of anticancer drugs and studied the release profiles with detail.

## Experimental

### Materials

Fumaric acid, PEGs, ethylene glycol, the initiator (Irgacure 500), DMSO-d_6_ and CDCl_3_ are all commercially available and were used as received from the suppliers. Paclitaxel and doxorubicin were kindly provided by Laboratorios Bioprofarma (Laboratorios Bagó, Buenos Aires, Argentina) and Lipomize S.R.L. (Santa Fe, Argentina). DMSO was distilled under vacuum and stored over molecular sieves (4 Å). THF was distilled from KOH.

### Methods

#### Synthesis and characterization of pre-polymers

Pre-polymers were obtained by direct poly-condensation reactions: to a 50 mL, two-necked, round bottomed flask equipped with magnetic stirring and a vacuum outlet were added 39.7 mmol (4.6 g) of fumaric acid and 39.7 mmol of the diol (ethylene glycol, di-ethylene glycol, PEG 200 or PEG 400). The reaction flask was heated at 165 °C for 3–8 h, depending on the pre-polymer, see Table 1, and then for 10 minutes under high vacuum (5 mmHg), at the same temperature. After cooling to room temperature, all pre-polymers were characterized as follows.

**Table 1:** Synthesis conditions and properties of pre-polymers

Pre-polymer	*t* a	*T*_g_ b	*T*_d_ b	*M*_n_ c	*M*_w_ c	DP	Monomer ratio^d^
EG–F	3.0	−54	370	350	620	2.8	1 : 1.02
dEG–F	4.5	−42	370	510	860	3.0	1 : 0.90
PEG_200_–F	6.0	−47	380	930	2680	3.3	1 : 1.03
PEG_400_–F	8.0	−52	380	770	2160	6.0	1 : 1.14

a Reaction time in h at 165 °C, followed by 10 min under high vacuum (5 mm Hg).

b In °C.

c In Da.

d Diol/fumaric acid ratio determined by ^1^H NMR.

*M*_w_ (weight-average molecular weight) and *M*_n_ (number-average molecular weight) were obtained by gel permeation chromatography analyses (GPC) using a Waters 1525 instrument equipped with a refractive index detector (Waters 2414) and a Waters Styragel HR4 + HR1 column. THF was used as elution solvent and to prepare 1 wt% pre-polymer solutions. The elution rate was 1 mL min^−1^ and polystyrene standards were used for the molecular weight calibration curves.

The degree of polymerization (DP) was calculated according to eqn (1).1
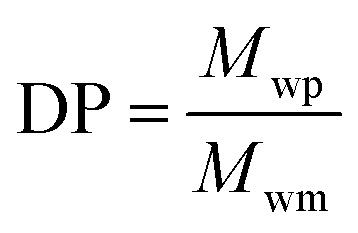
where *M*_wp_ is the *M*_w_ of the pre-polymer and *M*_wm_ is the *M*_w_ of the repeating unit.

^1^H NMR spectra were obtained with a Bruker Avance II 300 MHz spectrometer using DMSO-d_6_ (ethylene glycol fumarate (EG–F) and di-ethylene glycol fumarate (dEG–F) pre-polymers) or CDCl_3_ as solvent (PEG 200 fumarate (PEG_200_–F) and PEG 400 fumarate (PEG_400_–F) pre-polymers). The residual solvent signals (2.53 ppm for DMSO-d_6_, and 7.28 ppm for CDCl_3_) were used as reference.

EG–F and dEG–F: ^1^H-NMR (300 MHz, DMSO-d_6_): 6.5–6.8 (–HC=CH–, fumaric acid), 3.50 (O–C*H*_*2*_–C*H*_*2*_–O, PEG).

PEG_200_–F and PEG_400_–F: (300 MHz, CDCl_3_): 6.5–6.8 (–HC=CH–, fumaric acid), 3.50 (O–C*H*_*2*_–C*H*_*2*_–O, PEG).

Fourier transform infrared (FTIR) spectra were obtained with a Shimadzu 8201 PC apparatus.

The glass transition temperature (*T*_g_), melting temperature (*T*_m_) and/or crystallization temperature (*T*_c_) of pre-polymers were measured by differential scanning calorimetry (DSC Q2000, TA Instrument) at a heating rate of 10 °C min^−1^. Thermal stability was assessed by thermogravimetric analysis (TGA) a using a TGA Q500 (TA Instruments) equipment. Temperature scans were performed at a rate of 10 °C min^−1^ from room temperature to 600 °C. DSC and TGA experiments were performed under inert N_2_ atmosphere (flow: 40 mL min^−1^)

#### Preparation of photo-cured elastomeric films

Elastomers were prepared by the solvent casting technique to obtain a thin and uniform film: each pre-polymer was dissolved in THF and 1 wt% of Irgacure 500 was added. The solution was poured onto a glass slide and the solvent was left to evaporate for 12 h. Finally, the glass slides were exposed to UV light (350 nm) for different periods of time. 7 Actinic BL 8W (Philips) lamps were used in parallel and the samples were placed at a distance of 5 cm.

#### Characterization of photo-cured elastomeric films

Gel content was measured on films exposed for 30 minutes to UV light. Samples were cut in disc-shaped films of 1 cm diameter and 1 mm thick. A Soxhlet extraction apparatus and boiling ethanol were used for gel content determinations. After 24 h of extraction, samples were left to dry for 48 h at room temperature. Gel contents were obtained gravimetrically, according to eqn (2).2
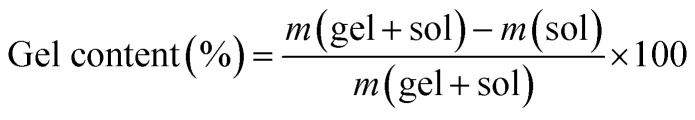
where *m*(gel + sol) is the total dry mass weighted before Soxhlet extraction and *m*(sol) is the soluble portion eliminated during the extraction process.

The kinetics of the photo cross-linking reactions were evaluated by UV-Vis spectroscopy in a Perkin Elmer Lambda 35 spectrophotometer. The THF solutions of the pre-polymers containing 1 wt% of Irgacure 500 were cast into a quartz slides and left to evaporate at room temperature under air. Then, UV-Vis spectra (190–400 nm) were recorded after exposure to UV irradiation for different periods of time.

*T*_g_, *T*_m_ and/or *T*_c_ of elastomers, as well as their thermal stability, were determined as described before.

Dynamic mechanical thermal analysis (DMTA) in tension mode was employed to investigate the rheological behavior of photo-cured elastomers. To this effect, films of 0.5 mm in thickness were tested under frequency sweeps (0.1–1.0 Hz at 30 °C) and temperature sweeps (30–300 °C, 5 °C min^−1^ of heating rate, and at 1 Hz).

Swelling indexes by hydration (eqn (3)) were obtained by immersing the samples in saline phosphate buffer (pH 7.4) at 37 °C: disc-shaped samples (0.8 cm × 0.1 cm) were cut from film sheets, placed in ethanol to remove the soluble fraction and left to dry. Samples were immersed in the buffer and the hydrated material mass was weighted after removing the excess of water from the surface at different periods of time, until water uptake was stable.3
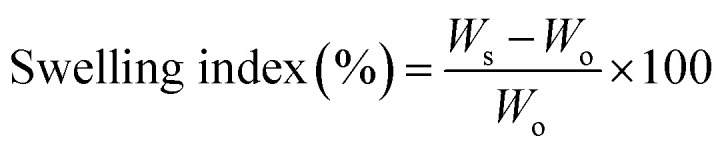
where, *W*_s_ is swelled mass and *W*_o_ is the initial dry mass.

#### Preparation of DOXO-loaded elastomers and their *in vitro* release

Films loaded with doxorubicin (DOXO) were prepared as follows. The pre-polymers dissolved in DMSO, containing 1 wt% of the initiator (Irgacure 500) and 2 wt% of DOXO, were casted onto a glass slide (2.0 cm × 2.0 cm) and the solvent was left to evaporate for 48 h at room temperature. Then, the pre-polymers containing the drug were exposed to UV light to obtain elastomeric films.

Each elastomeric film was cut into 1.0 cm × 1.0 cm samples with 0.1 cm of width and DOXO release was evaluated by immersing the samples in 10 mL of PBS buffer pH 7.4 and incubated at 37 °C under orbital stirring. 500 μl samples of the release medium were taken at different times for DOXO UV-Vis quantification, and replaced with fresh solution. A calibration curve was obtained by preparing standard solutions of DOXO in PBS buffer and absorbance at 495 nm was measured by UV-Vis spectrophotometry.

#### Preparation of PTX-loaded elastomers and their *in vitro* release

Films loaded with paclitaxel (PTX) were prepared in the same fashion as for DOXO, but using THF as solvent. PBS buffer (pH 7.4) containing 0.3% sodium dodecyl sulfate was used as release medium. Each piece of elastomer was immersed in 5 mL of PBS buffer under orbital shaking at 37 °C. Samples (500 μl) were taken at different times and replaced with fresh buffer solution. The amount of released PTX was determined by RP-HPLC following the same method that has recently been published.^17^

## Results and discussion

### Preparation of fumarate co-PEG pre-polymers

PEG-based fumarate pre-polymers can be prepared by the reaction of fumaryl chloride and PEG in the presence of triethylamine as HCl scavenger, or by Steglich esterification of fumaric acid and PEG, using DCC for the activation of the carboxyl group and DMAP as catalyst. In these reactions triethylammonium hydrogen chloride and di-cyclohexyl urea are formed as by-products, respectively. Therefore, PEG-based fumarate pre-polymers require extensive purification by re-crystallization if these synthesis methods are followed. We obtained the pre-polymers (ethylene glycol fumarate (EG=–F), diethylene glycol fumarate (dEG–F), PEG 200 fumarate (PEG_200_–F) and PEG 400 fumarate (PEG_400_–F)), by melt poly-condensation of the corresponding diols and fumaric acid (eqn (4)), which does not require the use of an additional organic base or a carboxylic group activator and neither an intermediate purification step.4
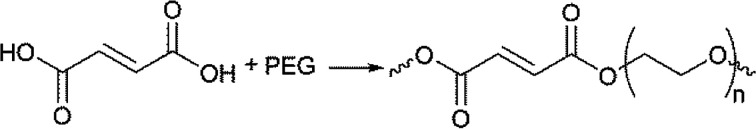
5
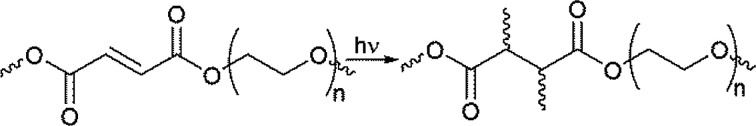


**Fig. 1:**
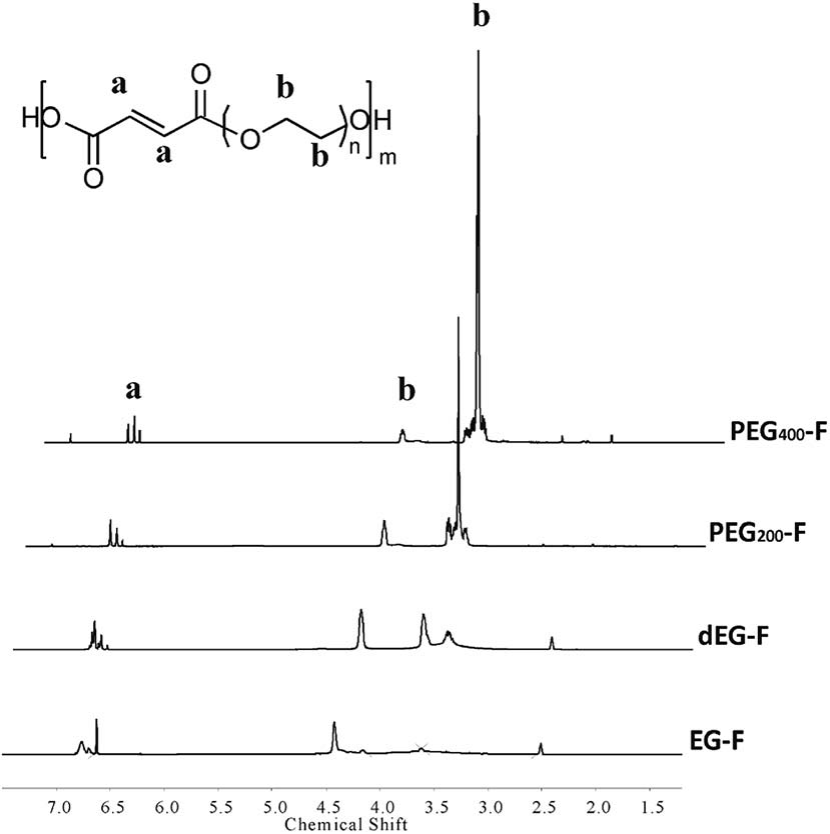
^1^H-NMR spectra for all pre-polymers.

We found that long reaction times produced solid polyesters, precluding their use for the preparation of elastomeric films in the second photo-curing step (eqn (5)). Therefore, the poly-condensation reactions were stopped at the times indicated in Table 1, at which the pre-polymers were obtained as highly viscous liquids (PEG_200_–F and PEG_400_–F, Fig. 1) or as pasty solids (EG–F and dEG–F, Fig. 1).

Table 1 summarizes the main characterization results of the pre-polymeric materials by ^1^H NMR, DSC and GPC. All pre-polymers were obtained as low molecular weight and poly-disperse materials (Table 1). The estimated degree of polymerization (DP) was around 3 for all pre-polymers, with the exception of PEG_400_–F (longest reaction time), which afforded a DP of 6. DSC scans showed low *T*_g_ values (around −50 °C) for all pre-polymers; while melting and crystallization temperatures were not registered within the evaluated temperature range (−90 °C to 60 °C). These results assure an amorphous state with high fluidity of all materials at room temperature. All pre-polymers showed an almost identical decomposition temperatures (*T*_d_) of 370–380 °C.

Given that the poly-condensation reactions were not performed in the presence of a radical polymerization inhibitor (radical scavenger), thermally-induced, radical cross-linking at the level of the double bond of fumaric acid could easily occur.

**Fig. 2:**
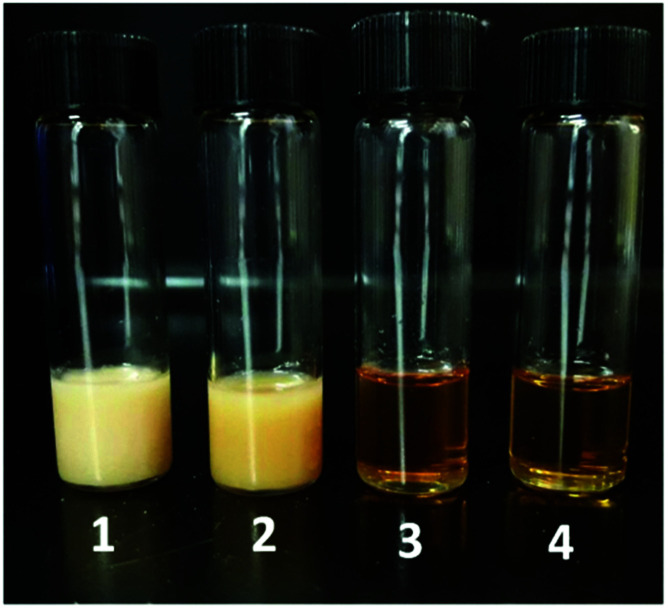
Photograph of EG–F (1); dEG–F (2); PEG_200_–F 200 (3) and PEG_400_–F (4).

Thus, to evaluate the degree of cross-linking at the unsaturated bond, and to further probe the structure of the materials, the pre-polymers were characterized by ^1^H NMR (Fig. 2). Integration of the signals that appear at around 3.50 and 4.50 ppm, corresponding to the ethylene glycol units, and the signals between 6.60 and 6.80, which belong to the double bond of fumaric acid, allowed us to estimate the actual monomer ratio (Table 1), and so, the cross-linking at the level of the double bond. As shown in Table 1, and within the experimental error, monomer ratios for all pre-polymers were close to 1, indicating that the radical polymerization of the double bond did not occur, at least significantly, for none of the materials.

**Fig. 3:**
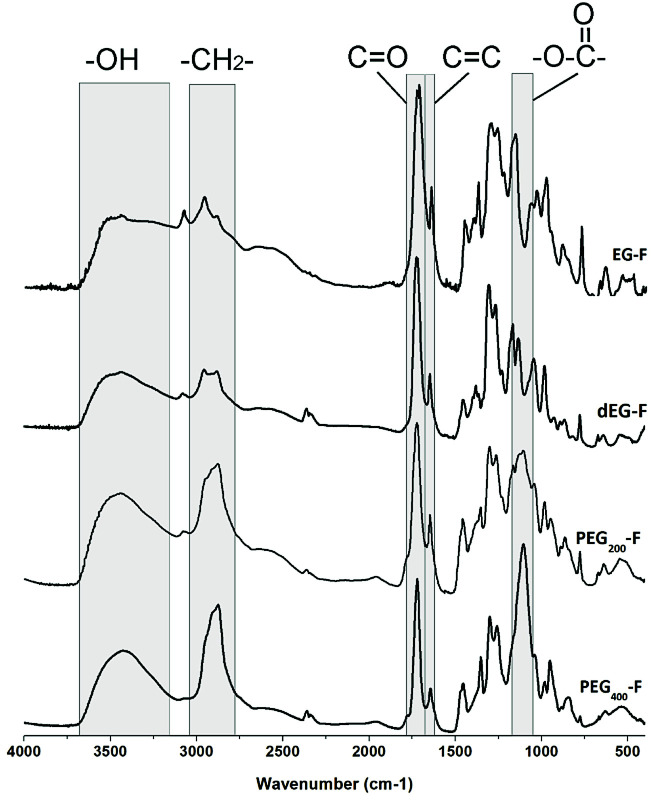
FT-IR spectra of EG–F, dEG–F, PEG_200_–F and PEG_400_–F pre-polymers.

FTIR spectra of pre-polymers, which are presented in Fig. 3, showed peaks at 1730 cm^−1^ (C=O stretching), 1640 cm^−1^ (C=C stretching) and 3500 cm^−1^ (–OH bend). The intensity of –OH peak suggests that the thermal poly-esterification reactions were not complete.

### Preparation and characterization of photo-cured polymers

Irgacure 500 (a commercially available mixture of benzophenone and 1-hydroxy-cyclohexyl-phenyl-ketone) was used at a concentration of 1% wt for the photo cross-linking of EG–F, dEG–F, PEG_200_–F and PEG_400_–F pre-polymers. Thus, neat pre-polymers were casted on glass slides and then exposed to UV light (350 nm) in a low-power photo-reactor. For dEG–F pre-polymer, we found out that the slightly opaque, pasty, initial material (Fig. 1) was transformed to a completely transparent and clear polymeric film after UV irradiation. On the contrary, EG–F pre-polymer yielded a white and brittle polymer, and not an elastomeric film. For this reason this materials was discharged and not further studied.

**Fig. 4:**
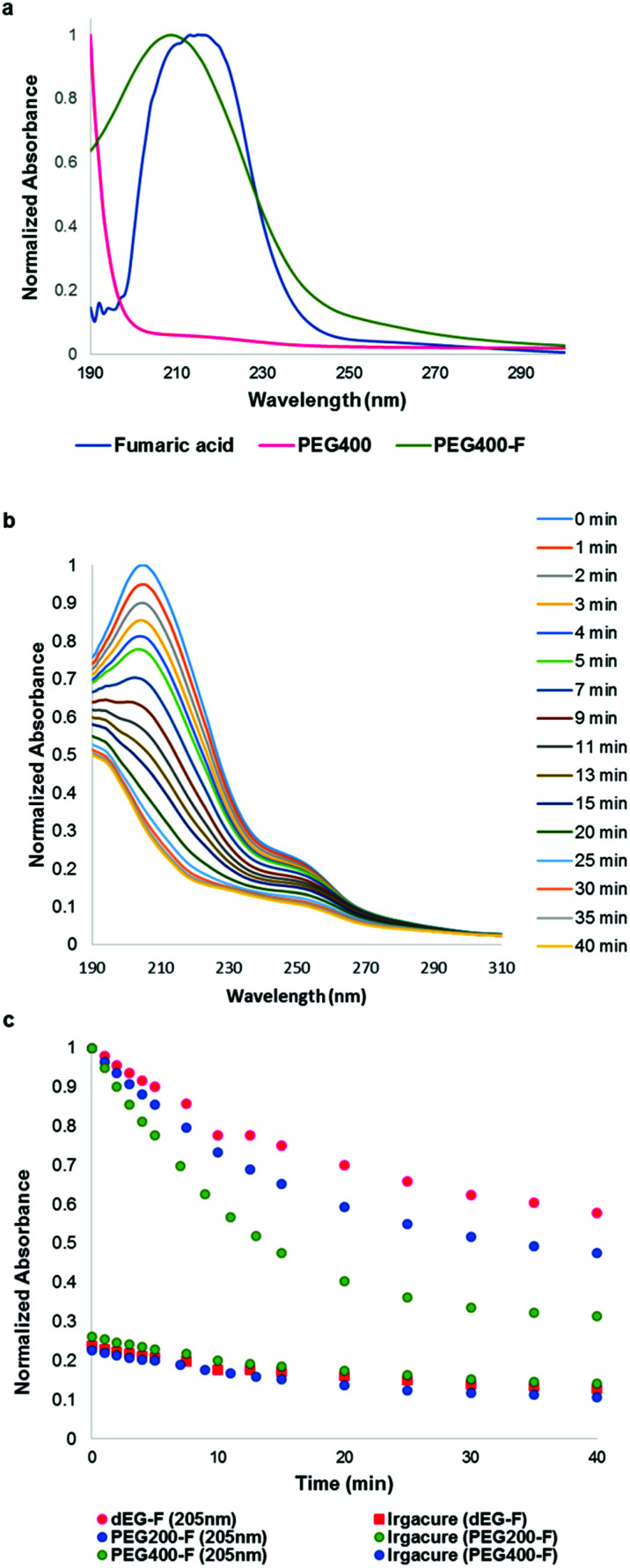
UV-Vis analysis of photo-curing process. (a) UV-Vis spectra of reagents and pre-PEG_400_–F 400 (b) UV-Vis spectra after different UV irradiation times of elastomer PEG_400_–F (c) peak absorbance (205 nm for pre-polymers and 250 nm for initiator) *vs.* irradiation time.

In order to understand with more detail the evolution of the photo-curing process, the UV Irradiation of quartz glass slides supporting the elastomeric films, was stopped at different times and analyzed by UV-Vis spectrophotometry (Fig. 4).

As reference, Fig. 4a shows UV-Vis spectra of the monomers and pre-PEG_400_–F. Fumaric acid has an absorption peak at 215 nm, associated to the doubly conjugated C=C bond, while the absorption peak of oligo-PEG_400_–F is slightly shifted to shorter wavelengths (205 nm), probably due to the esterification reaction (Fig. 4a). The *λ*_max_ of Irgacure 500 is located at 250 nm, as indicated by the supplier of the initiator mixture. Accordingly, the disappearance of the peak at 205 nm can be associated to the cross-linking of the pre-polymer, at the level of the double bond, to yield a 3D elastomeric network (Fig. 4b). In the same fashion, the disappearance of the peak at 250 nm can be attributed to the consumption of the initiator mixture (Fig. 4b). We found that the rate of consumption of Irgacure 500 was low and it was almost the same for all pre-polymers (Fig. 4c).6
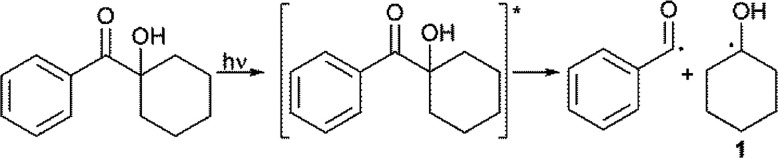
7
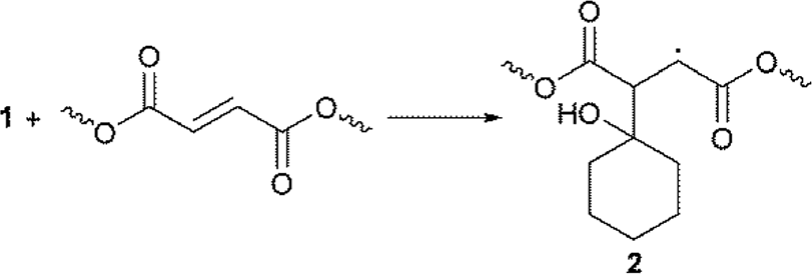


It is well known that 1-hydroxy-alkyl-phenyl-ketones, such as 1-hydroxy-cyclohexyl-phenyl-ketone, can efficiently promote light-induced cross-linking processes (eqn (6)). Thus, after homolysis, α-oxo radical 1 should add to a double bond of PEG–fumarate to propagate the cross-linking reaction, forming addition product 2, in the same fashion as has been proposed for other related systems (eqn (7)).^18^

The hydrogen atom abstraction from one oxy-ethylene unit of PEG, by the triplet-state of benzophenone to yields α-oxo-radical 3 and bis-benzhydryl radical 4 (eqn (8)), can also account for the initiation reaction. Radical 3 should then add to the unsaturated bond of a fumaric acid residue to yield the α-acyl radical 5 (eqn (9)), which can propagate the cross-linking process by sequential addition reactions. Of course, hydrogen atom transfer from radical 4 to radicals of the type of 5 can act as termination steps, mainly at the end of the cross-linking reaction, yielding benzophenone, which in fact can act as a photo-catalyst.^19^ This observation may explain the un-complete consumption of the initiator mixture.8
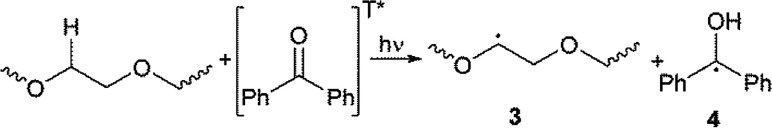
9
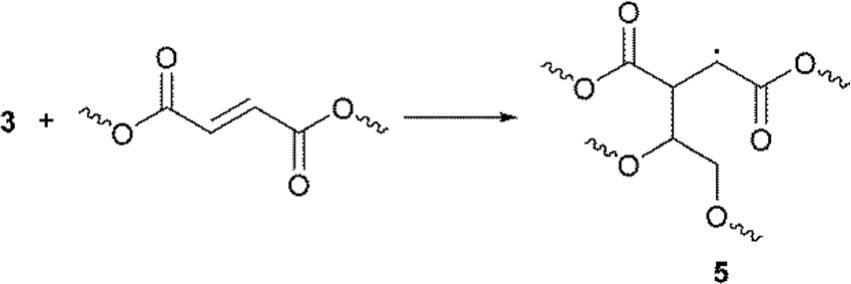


For the surface functionalization of PEG micro-particles, a two-step benzophenone-mediated method has been reported.^20^ In the first step the PEG particles are grafted with benzophenone under photo-stimulation, in a process that involves homo-coupling of radicals 3 and 4 (eqn (10)). After washing, in the second step of the functionalization protocol, the benzophenone-grafted particles are mixed with acrylic monomers and polymerized to yield 7 under UV irradiation (eqn (11)). Probably, the addition reaction of eqn (9) involves an intermediate homolysis step of 6.10
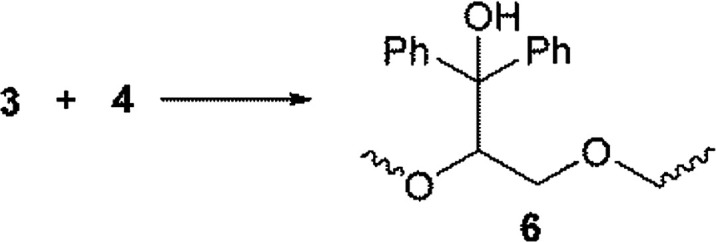
11
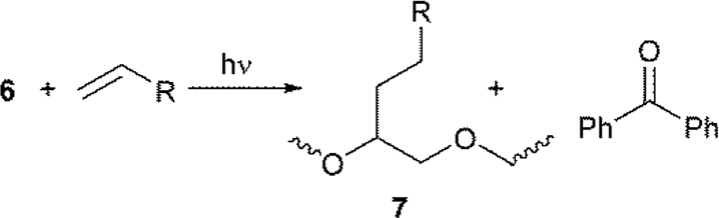


Due to the low LUMO energy expected for fumaric acid residues, and given that the unsaturated radical acceptor (*i.e.*, the double bonds of fumaric acid) are present from the beginning of the radical cross-linking reaction, the two-step mechanism depicted in eqn (10) and (11) appears to be unlikely for our system, and probably, for benzophenone, the mechanism represented by eqn (8) and (9) operates under the experimental conditions used herein, in the same fashion as has been proposed by the same authors for the one-step functionalization of PEG micro-particles.^20^ A similar mechanism has recently been proposed for the benzophenone promoted photo cross-linking of acrylates.^21^

**Fig. 5:**
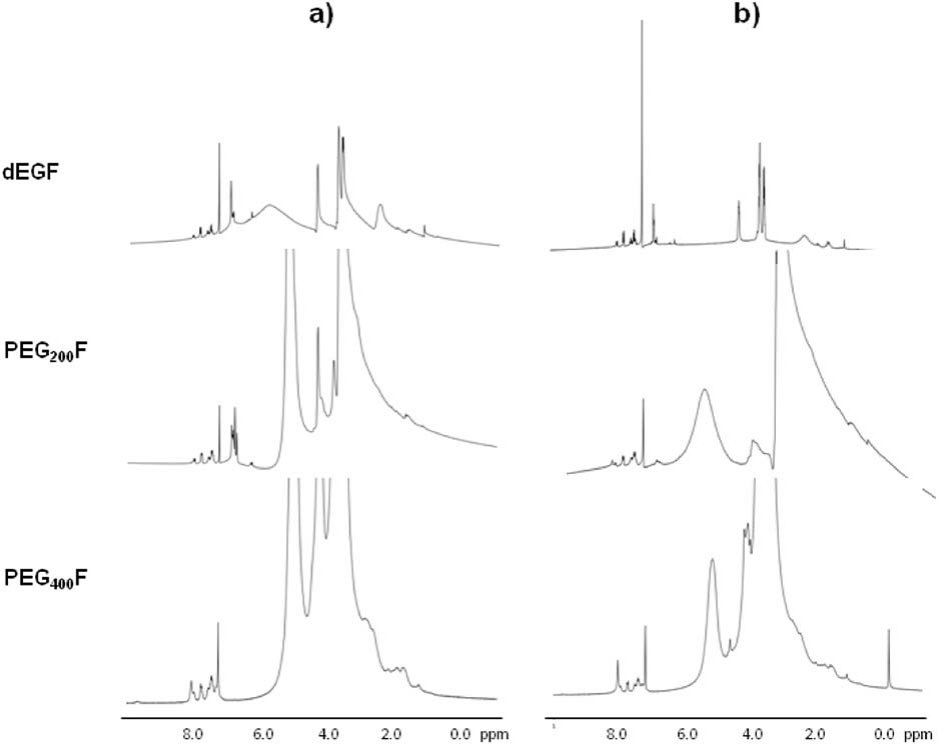
^1^H NMR of swelled polymers after 30 minutes of irradiation (a) followed of heating at 110 °C for 3 h (b).

The rate of decrease of the band at 205 nm, associated with consumption of the double C–C bond, appears to be higher for PEG_400_–F than for PEG_200_–F and dEG–F (Fig. 4c). We also visually observed that glass-transition points for dEG–F and PEG_200_–F were reached faster than for PEG_400_–F. One possibility to explain these results relays on the fact that as the molecular weight of the (PEG) diol increases, the double C–C bonds, which act as cross-linking points, are further separated by more flexible oxy-ethylene units, allowing for higher molecular movement at the same cross-linking degree. In other words, as the length of the PEG chain increases, at the same cross-linking degree, the material is more fluid, allowing for higher rates of double bond consumption. Of course, from UV-Vis spectra shown in Fig. 4b complete consumption of the double bonds of fumaric acid residues cannot be assumed. Therefore, with the aim of evaluating whether or not there were still available un-reacted double bonds after 30 minutes of irradiation, the elastomers were swollen in CDCl_3_ and analyzed by ^1^H NMR (Fig. 5a). After the ^1^H NMR experiments, and with the goal of evaluating if cross-linking of the double bonds is possible under thermal conditions, the materials were dried at room temperature for 48 h and then heated at 110 °C for 3 h. After swelling with CDCl_3_ these thermally-treated elastomers were also studied by ^1^H NMR (Fig. 5b).

The signals located after 7.5 ppm are assigned to the aromatic rings of the photo-initiator mixture, while those at around 6.5 ppm are assigned to the double bonds of fumaric acid residues. Although the results shown in Fig. 5 are not conclusive, for PEG_400_–F it seems that no double bonds remains after the photo-curing process and, of course, after heating at 110 °C. In clear contrast, the spectra of PEG_200_–F and dEG–F show remaining vinylic proton signals after 30 minutes of irradiation (Fig. 5a) in agreement with UV-Vis spectrophotometry analyses. The decrease of the intensity of double bond signals obtained upon heating at 110 °C suggest that further thermal curing of PEG_200_–F and dEG–F is possible after the irradiation time (Fig. 5b). These experimental results do not contradict those obtained for the thermal stability (cross-linking) of the pre-polymers (Table 1 and Fig. 2), since the syntheses of the pre-polymers were performed in the absence of the initiator. Although it is not clear how Irgacure 500, ascribed as a purely photochemical initiator, can further induce the radical cross-linking, the thermal homolysis of 1-hydroxy-cyclohexyl-phenyl-ketone (eqn (6)) appears as one possibility to explain this surprising results.

**Fig. 6:**
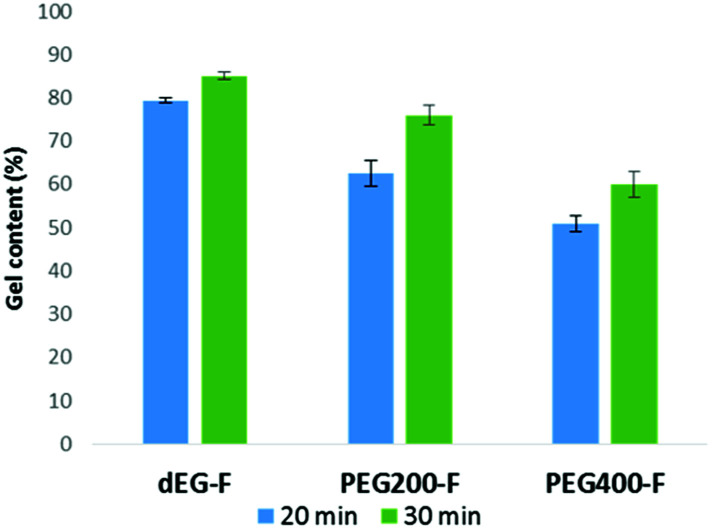
Gel content of dEG–F, PEG_200_–F and PEG_400_–F photo-cured for 20 and 30 minutes.

By Soxhlet extraction with hot ethanol, we measured the gel fraction content on the photo-elastomers irradiated for 20 and 30 minutes (Fig. 6). The insoluble fraction values of the photo-cured films depend on the ethylene glycol content of the material. While around 80% of the photo-cured dEG–F film was insoluble, there were still a high sol fractions for elastomers PEG_200_–F and PEG_400_–F, suggesting that for these last two materials a higher cross-linking is still possible, which would allow to cover a wider spectra of mechanical properties. From Fig. 6 it can also be noted that the gel content increases with the irradiation time. Thus, higher values were obtained after 30 minutes of irradiation than for 20 minutes. These results further support the hypothesis that the consumption of double bonds of fumaric acid residues was not completed. For the purposes of this work, we decided to select the materials obtained after 30 minutes of irradiation.

**Fig. 7:**
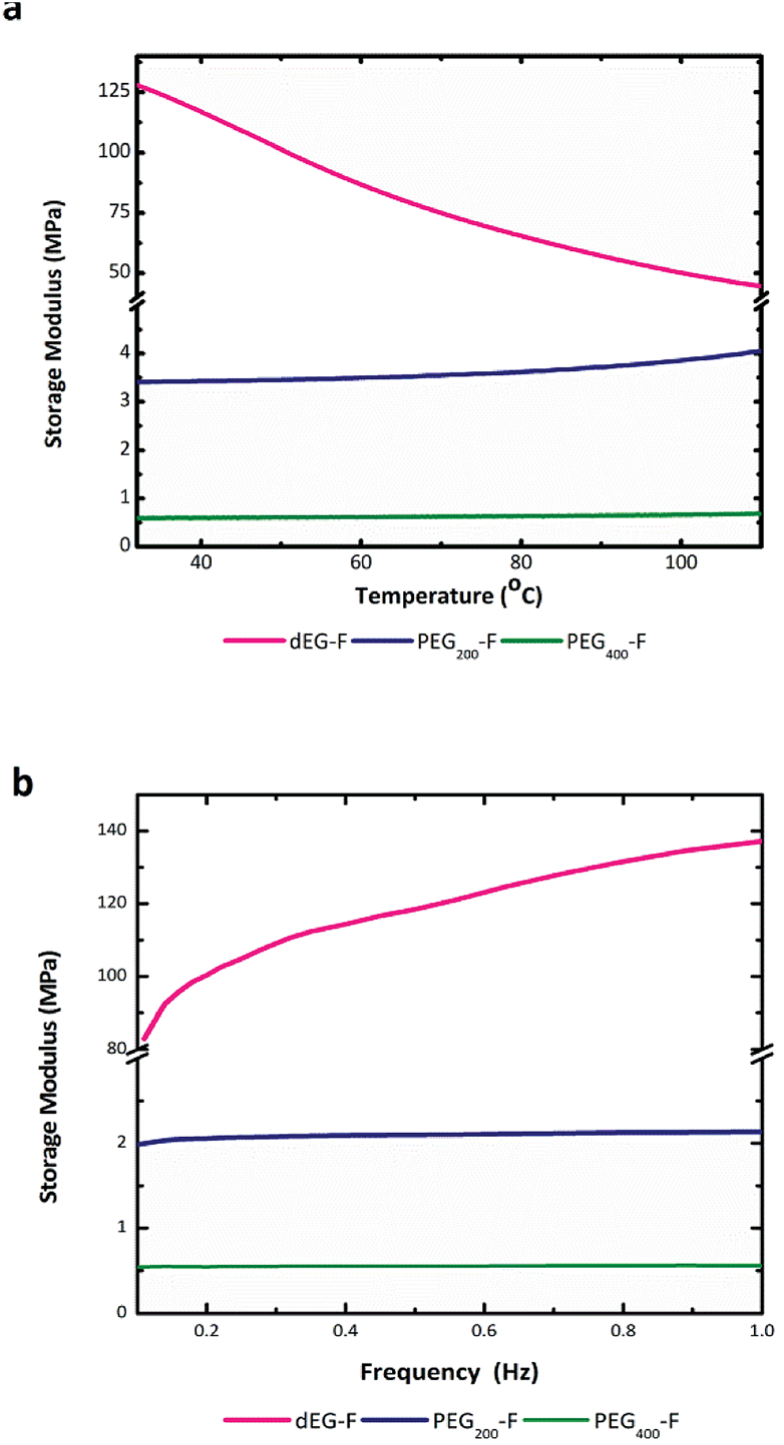
DMTA analysis of film samples of dEG–F, PEG_200_–F and PEG_400_–F. Frequency (a) and temperature (b) sweeps.

DMTA analyses were performed on the materials photo-cured for 30 minutes by applying thermal heating rate of 5 °C min^−1^ (Fig. 6a) and frequency sweep of 0.1–1 Hz (Fig. 7b). The storage modulus (*G*′) increases as the length of oligo-ethylene glycol chain decrease (*i.e.*, films stiffen). These results further supports the trend that films with increasing cross-linking density were obtained upon reduction of the length of the oxy-ethylene chain length. Note that photo-cured dEG–F elastomer, which presented the highest gel content value, presented a decreasing *G*′ with temperature, as consequence of material soften. On the other hand, PEG_200_–F and PEG_400_–F showed that *G*′ modulus tends to increase with temperature, especially over 100 °C, indicating an increase of the cross-linking degree. This behaviour is in accordance with the high soluble fractions values (Fig. 6), which anticipated that, at these curing times, the materials might still contain relatively short un-cross-linked polymeric chains, which are soluble in the solvent used for the extraction. In addition, and taking into account the low *M*_w_ values obtained for all pre-polymers after the thermal poly-condensation reaction (Table 1), it is likely that during DMA analyses the thermal curing of un-reacted OH and C(O)OH groups also occurs. Moreover, the thermal radical cross-linking, at the level of remaining double bonds of fumaric acid residues, is also feasible during the DMTA analyses, as it was previously observed for dEG–F and PEG_200_–F (Fig. 5b).

Table 2 shows the thermal properties of all photo-cured elastomers. All materials showed almost identical decomposition temperatures (*T*_d_) of around 400 °C. In addition, the initial 10% mass loss due to decomposition (*T*_10%_) occurred for all elastomers at around 200 °C. These results indicate that these polymers could easily be sterilized using standard high-pressure vapor techniques.

**Table 2:** Thermal properties of photo-cured polymers^a^

Material	*T*_10%_ (°C)	*T*_d_ (°C)	*T*_g_ (°C)
dEG–F	198	393	−20
PEG_200_–F	182	394	−39
PEG_400_–F	227	393	−44

a *T*_10%_ is the decomposition temperature for 10% mass loss.

**Fig. 8:**
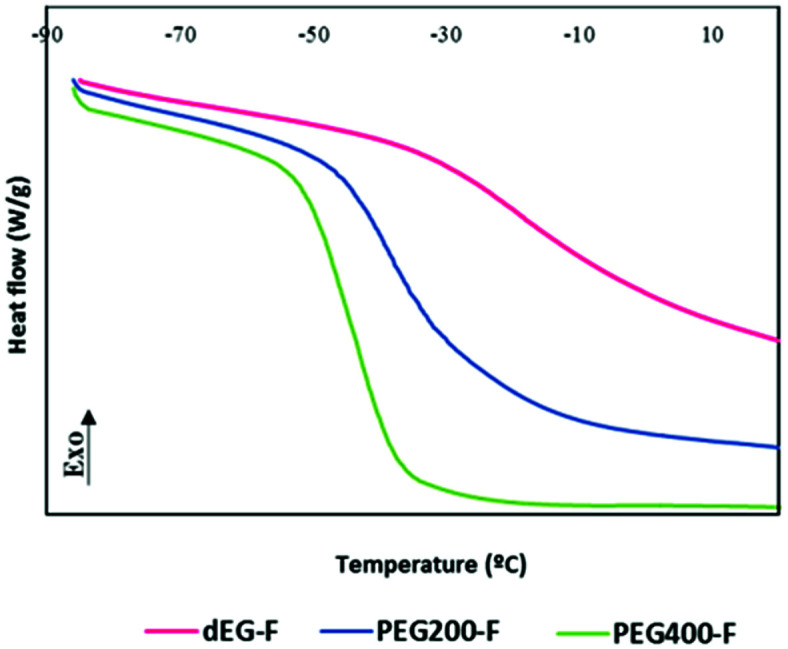
DSC scans of elastomeric films of dEG–F, PEG_200_–F and PEG_400_–F.

*T*_g_ values of elastomers tend to decrease when the molecular weight of the PEG co-monomer increases. Thus, *T*_g_ temperatures of −20, −39 and −44 °C were registered for dEG–F, PEG_200_–F and PEG_400_–F, respectively. As indicated above, this behavior is probably related to a higher chain mobility provided by the increase of the molecular weight of the diol monomer. From Fig. 8 it is clear that the glass transition state for dEG–F occurred at a wider temperature range. This result is consistent with the findings in DMTA analyses, where *G*′ decreases as the temperature increases.

**Fig. 9:**
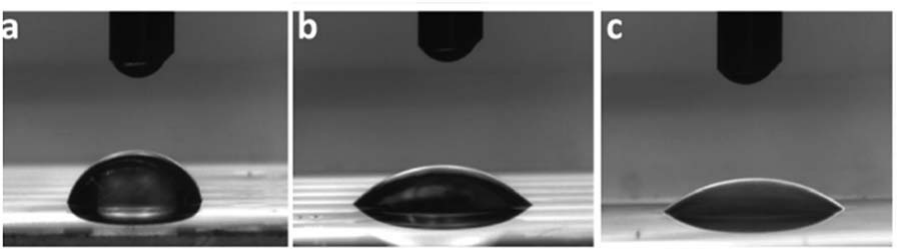
Contact angle measurements photographs, of dEG–F (a), PEG_200_–F (b) and PEG_400_–F (c).

The film surface hydrophilicity of all materials was evaluated by contact angle measurements using water as dispensed solvent (*θ*). It was found that as the molecular weight of PEG increases, the surface hydrophilicity also increases. Fig. 9 shows pictures of the dispensed drops on the films surfaces. Thus, *θ* values of 77.4 ± 3.3°, 44.2 ± 4.0° and 40.1 ± 5.0° were obtained for dEG–F, PEG_200_–F and PEG_400_–F, respectively.

**Fig. 10:**
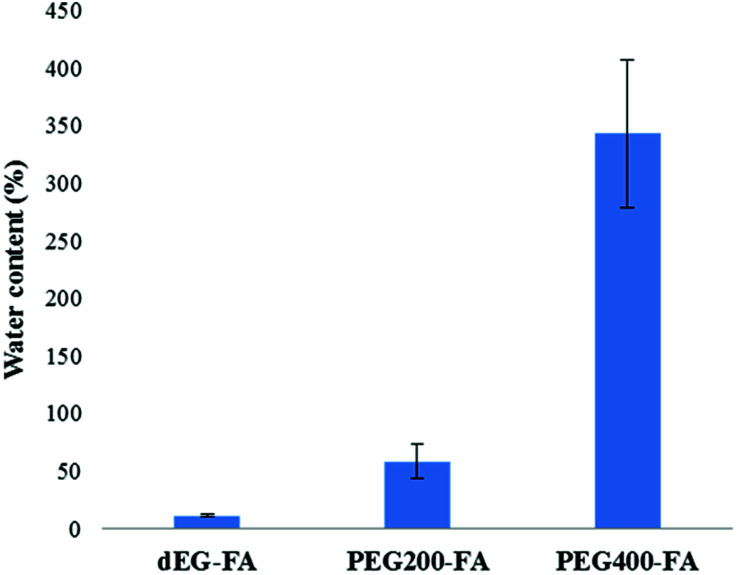
Swelling indexes of elastomeric films due water uptake.

**Fig. 11:**
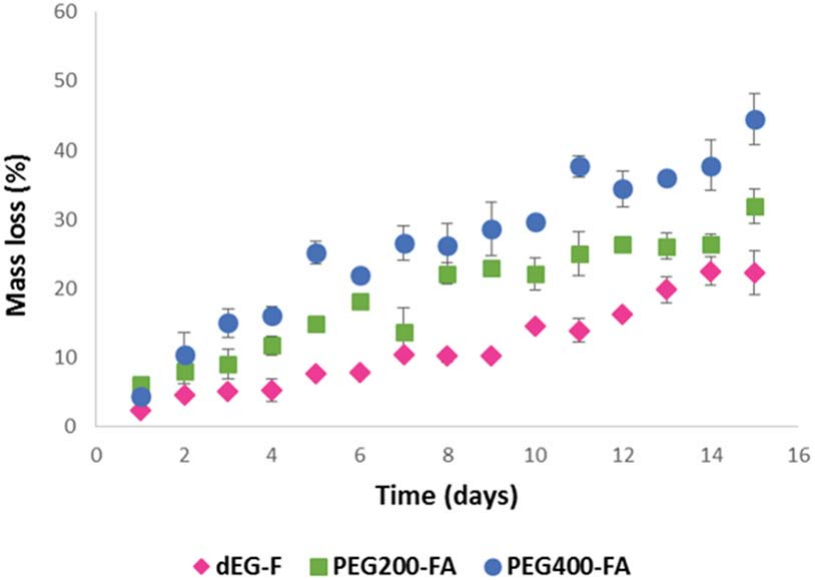
Hydrolytic degradation of photo-cured films.

With the aim of understanding the behavior of the elastomeric films in aqueous environment, swelling indexes and hydrolytic degradation profiles were obtained (Fig. 10 and 11).

The swelling indexes due water uptake for the three elastomers were significantly different, as shown in Fig. 10. The highest swelling index was obtained for PEG_400_–F (343 ± 64%), for which a high deformation was also observed. On the contrary, dEG–F presented a much lower swelling index value (11 ± 1%), while it almost maintained its original size. These results agree with the higher hydrophilicity and less cross-linking density anticipated for PEG_400_–F. As expected, the swelling index of PEG_200_–F was intermediate with those of dEG–F and PEG_400_–F.

The swelling behavior of the PEG–fumarate materials depends on their cross-linking degree and, importantly, the molecular weight of the PEG. For example, the seminal work of Mikos showed that a swelling ratio of around 600% was obtained for a cross-linked PEF prepared with a PEG of 1 kDa, using a fumaric acid (FA)/PEG ratio of 0.9, and 1% w/w of the initiator.^22^ A swelling ratio of around 1600% was obtained for the material prepared using identical AF/PEG molar ratio and initiator concentration, but employing a PEG of 4.6 kDa. Even higher swelling ratios can be expected for other closely related materials prepared in other related works, since it is usual to employ PEGs of higher molecular weights (10 kDa most commonly). In our work the highest swelling ratio (343%) was obtained using PEG of 400 Da (swelling ratios of around 10 and 50% were reached with DEG and PEG_200_ respectively). Thus, it is clear that our materials cannot be considered hydrogels.

As expected, the most hydrophilic elastomer (PEG_400_–F) afforded the fastest aqueous degradation profile, reaching almost 50% of mass loss after 15 days (Fig. 11). In contrast, dEG–F, which is less hydrophilic and has the highest cross-linking density network, presented a mass loss of around 20% for the same time spam.

### Anti-cancer drugs delivery

**Fig. 12:**
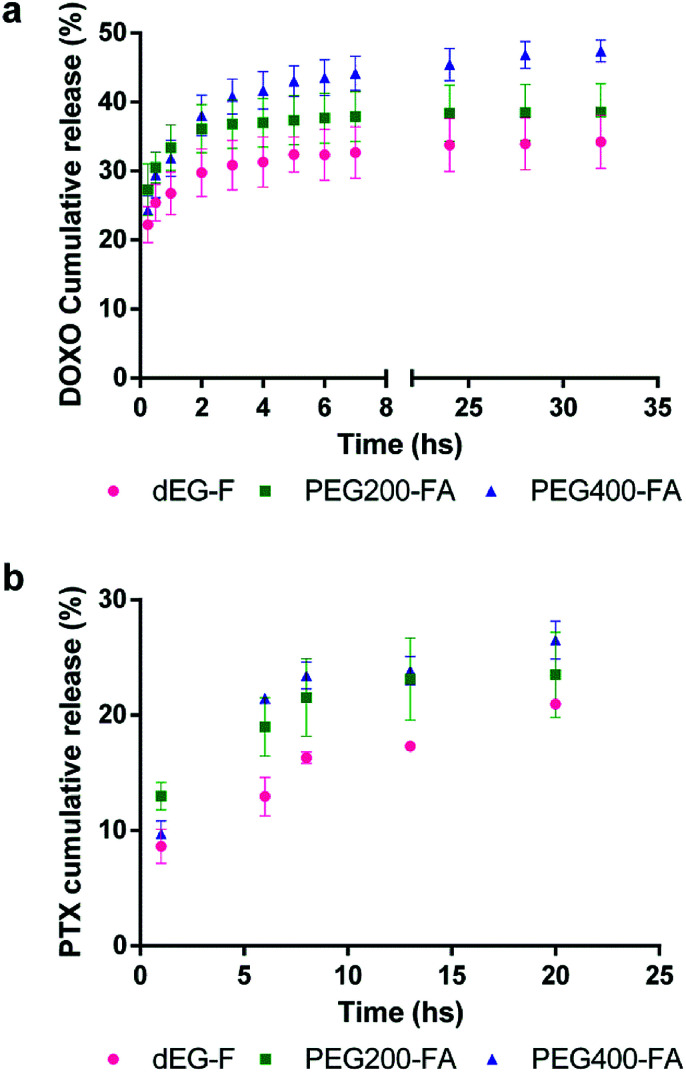
Cumulative release profiles from dEG–F, PEG_200_–F and PEG_400_–F. (a) DOXO and (b) PTX release.

With the goal of evaluating the potential utility of the new elastomers, we decided to evaluate the single-step construction of drug release systems using hydrophilic (DOXO) and hydrophobic (PTX) anti-cancer drugs. Thus, solutions containing the pre-polymers, the initiator and the drugs were casted onto glass slides and the solvent was left to evaporate at room temperature. After irradiation for 30 minutes with low power UV lamps, we were delighted to find that the drugs did not interfere with cross-linking process and that loaded elastomeric films were obtained in all cases. We were particularly surprised to see that even DOXO, which has an hydroquinone ring, was also able to afford a drug-elastomer construct. The polymeric drug-eluting systems were incubated at 37 °C in PBS buffer pH 7.4, and the drug release profiles are shown in Fig. 12.

DOXO release profile showed a marked burst phase, whose intensity depended on the hydrophilic/hydrophobic nature of material. PEG_400_–F, showed a very pronounced burst release of DOXO (Fig. 5a). This release profile probably results from the combination of three factors; namely, the hydrophilic character and the high water uptake of this elastomer, and the polarity and water solubility of the drug. For all materials, the burst release phases occurred during the first 6 h of incubation. At this time, the DOXO released for dEG–F, PEG_200_–F and PEG_400_–F was 32, 38 and 44%, respectively (Fig. 12a). This results match with those previously reported, and it has been recognised that to achieve controlled release of hydrophilic and positively charged DOXO, the PEG–fumarate oligomers should be co-polymerized with negatively charged sodium methylmethacrylate.^23^ On the other hand, PTX release showed a less pronounced burst release, probably due to the hydrophobic nature of the drug. Fast release occurred during the first day of incubation, which slowly decreased until the second week of incubation, when the release per day remained stable (Fig. 12). These slow release profiles of PTX indicate that the materials are interesting candidates for sustained release in long-term treatments.

## Conclusions

Following a two-steps approach, in this work we obtained a new set of elastomers based in oligo-ethylene glycol and fumaric acid. The synthesis of the materials involved a first thermal poly-condensation reaction to yield poly-ester pre-polymers, which were cured under mild conditions using low power UV lamps in the presence of a suitable radical photo-initiator. While ^1^H NMR experiments suggest that linear polyester pre-polymers are obtained almost exclusively in the first thermal reaction, a detailed UV-Vis spectroscopic study indicates that under irradiation the cross-linking process happens mainly at the level of the double bonds of fumaric acid monomers. In addition, we found that radical curing of the conjugated double bonds could, to some extent, also occur under thermal conditions in the presence of the radical initiator, which would contribute to explains the results of the TGA and DMTA studies. The new elastomers, which were fully characterized by several methods, were evaluated for construction of eluting system of hydrophilic and hydrophobic anti-cancer drugs (doxorubicin and paclitaxel). Thus, while the materials provided conventional release of hydrophilic DOXO, promising sustained release profiles were achieved for hydrophobic PTX.

## Conflicts of interest

There are no conflicts to declare.

## Supplementary Material
